# Manuka Essential Oil Triggers Apoptosis and Activation of c-Jun N-Terminal Kinase in Fibroblasts and Fibrosarcoma Cells

**DOI:** 10.3390/molecules29215168

**Published:** 2024-10-31

**Authors:** Noa I. Bass, Mruga Y. Parekh, Prabodh Satyal, Subah Soni, Jive A. Jacob, James P. Mack, Dorothy E. Lobo

**Affiliations:** 1 Department of Biology, Monmouth University, West Long Branch, NJ 07764, USAmack@momouth.edu (J.P.M.); 2 Aromatic Plant Research Center, Lehi, UT 84043, USA

**Keywords:** essential oils, manuka, JNK, apoptosis, fibrosarcoma, fibroblast

## Abstract

Manuka essential oil has long been used in traditional medicine, though the effects of the oil on cancer cells have limited studies. The goal of this project was to treat cancer cell lines with manuka essential oil at different concentrations and to ascertain the effects on the cell proliferation of normal fibroblast (CUA-4) and on fibrosarcoma (HT-1080) cells. Cell lines were grown on 24-well plates, and subconfluent cultures were treated with varying concentrations of manuka oil for 24 h. The effect of the oil on proliferation and viability was measured through direct cell counting using trypan blue dye exclusion and through the use of an MTT assay. As the concentration of oil increased, proliferation of all cell lines tested decreased with increasing dosage, concurrently with a decrease in MTT activity. To determine if the decrease in cell numbers observed from manuka oil treatment is the result of apoptosis, PARP cleavage assays were performed, confirming that the treatment caused apoptosis in both normal fibroblasts and fibrosarcoma cells. The stress-activated MAPK protein, JNK, was activated by manuka essential oil treatment, occurring synergistically with a decrease in MKP-1 expression.

## 1. Introduction

Essential oils (EOs) are organic, volatile liquids, mainly composed of members of the terpene family, in addition to monoterpenes, sesquiterpenes, and phenolic compounds [1]. EOs have been used for centuries by many cultures for domestic use as well as for medicinal purposes. Ancient Egyptians used aromatic oils for cosmetics dating back to 4500 B.C., with applications then expanded to Chinese and Indian medicine between 3000 and 2000 B.C. [2]. Oils and aromatic plants became a part of culinary, religious, and medicinal culture in ancient times. For example, juniper was burned in Tibetan temples in purification rituals, and the ancient Indian *Rig Veda* text describes the use of herbs for religious and therapeutic means. As distillation and the modern chemistry of oils evolved, knowledge of the chemical components of the oils emerged, and the concepts of aromatherapy arose [2,3]. EOs can be distilled from various parts of plants, including flowers, seeds, stems, roots, leaves, and bark. Today, in addition to their use in perfumes and culinary applications, there is increasing interest again in potential medicinal benefits from EO use. EOs are found in several types of aromatic plant cells and can be extracted using techniques such as steam distillation, solvent extraction, and hydrodistillation [2]. The duration of extraction can vary depending on the contents of the oils; differences in the extraction procedure can influence the chemical profile of the oil [2]. The multiple components of the oil may work synergistically to provide its bioactive properties.

The manuka tree, *Leptospermum scoparium*, is a member of the *Myrtaceae* family, and is a shrub that grows in New Zealand and Australia. In New Zealand, manuka has been traditionally used for a variety of applications, including mouthwash, pain relief, and the treatment of skin conditions [4]. The *Myrcia* family of plants, reviewed by Cascaes et al. [5], is a large family of flowering trees and shrubs which have leaves, bark, stems, and flowers that have been used in traditional medicine. Manuka essential oil, mainly obtained from the bark and seeds, has been used for centuries to treat wounds, infection, inflammation, and pain [6]. There is limited knowledge of the effects of manuka essential oil on the growth and proliferation of cancer cells. According to the American Cancer Society, the incidence rate of 6 out of the top 10 cancers in the United States has been increasing, with over 600,000 deaths from cancer projected to occur in 2024 [7]. The increased incidence of cancer, and the propensity for drug resistance, has caused a continuous demand for the search for new therapies. Many natural products, including plants, have been investigated for potential anti-cancer activity. Paclitaxol, vincristine, and etoposide are three examples of cancer chemotherapy agents that were first identified from plants [8]. Therefore, plant essential oils serve as a potential resource for such compounds and should be investigated.

As the metabolic products of plants, EOs confer survival advantages to plants in their unique habitats, including protection against pathogenic bacteria [4,9]. Less is known about the potential applications of essential oils for human health. More recently, attention has been given to the other potential health benefits, including anti-inflammatory and anti-carcinogenic effects, of various essential oils [2]. The treatment of the human leukemia cell line THP-1 with lipopolysaccharide (LPS) and manuka EO decreased the release of TNF-α [10]. Similarly, manuka EO has been found to reduce skin inflammation following UV-B exposure through suppressing the induction of the cytokines IL-1β and TNF-α [6]. This work examined the effect of manuka essential oil on the proliferative capacity and survival of normal human fibroblasts and fibrosarcoma cells. Treatment with manuka essential oil decreased the proliferation of these cells, activated c-Jun N-terminal kinase (JNK), and resulted in apoptosis.

## 2. Results

### 2.1. Composition of Manuka Essential Oil

**Figure 1:**
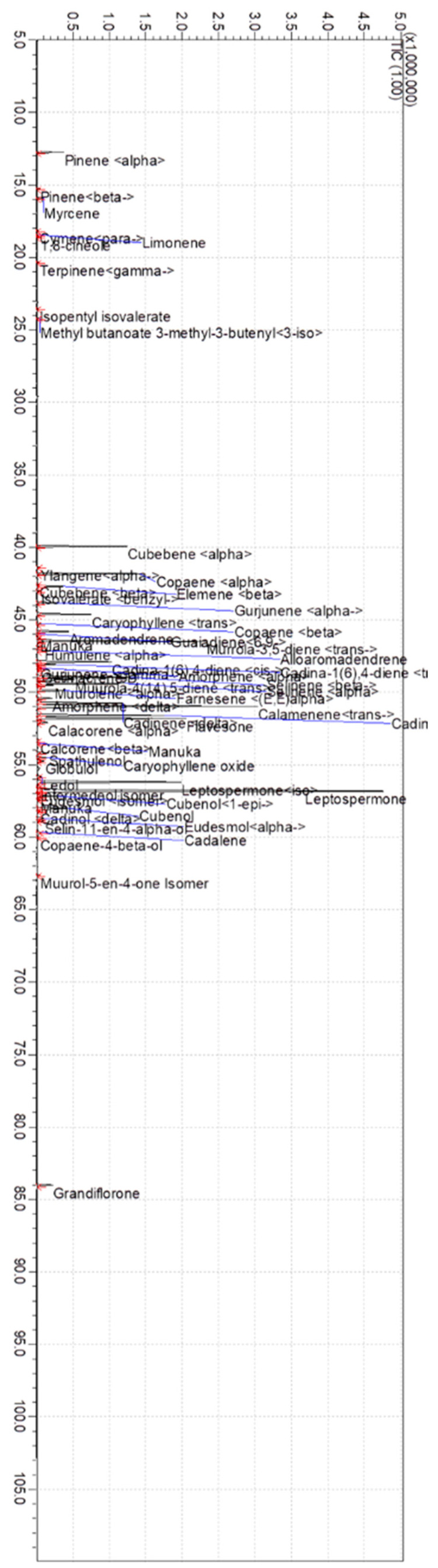
Chromatogram of manuka essential oil. Range of chemical constituents is indicated.

**Figure 2:**
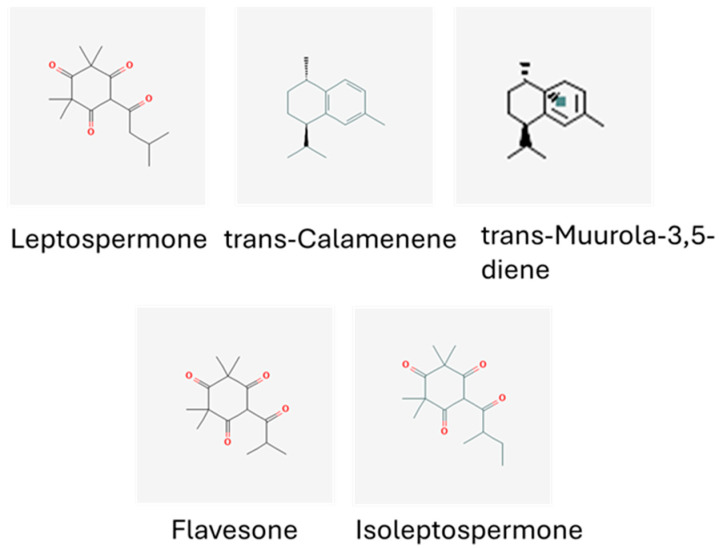
The major constituents of manuka essential oil. The major constituents extracted in this oil include leptospermone, trans-calamenene, trans muurola-3, 5-diene, flavesone, and isoleptospermone [11].

The chemical composition of manuka essential oil is shown in Figure 1 and Table 1. The triketones leptospermone (19.25%) and E-calamenene (11.56%) were found in the most abundance. Additionally, muurola-3,5-diene (6.95%), flavesone (6.46%), and isoleptospermone (6.15%) were also prominent (Figure 2) [11].

### 2.2. Manuka Essential Oil Decreased Proliferation and Viability

**Figure 3:**
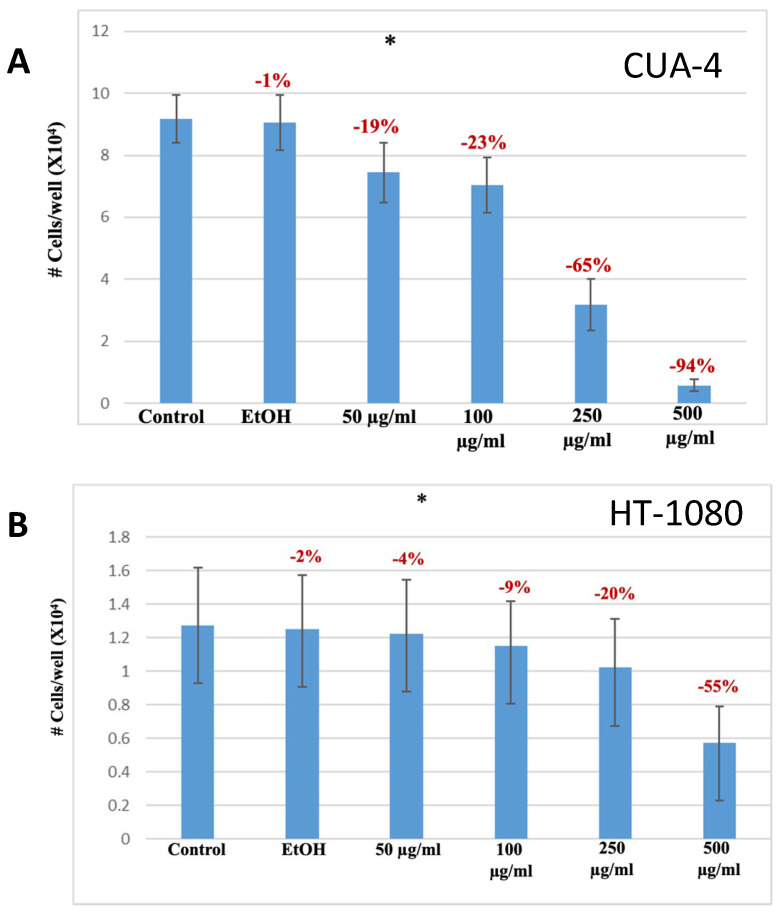
Manuka treatment decreased proliferation. The indicated cells were plated into 24-well plates and treated for 24 h with the indicated concentrations of manuka essential oil. Controls were left untreated (“control”) or were treated with ethanol (vehicle for oil dilution) (“EtOH”). (**A**) CUA-4 cells. * results were the average of 20 independent experiments and were found to be significant using ANOVA (α = 0.005). (**B**) HT-1080 cells. * results were the average of 10 independent experiments and were found to be significant using ANOVA (α = 0.05).

To ascertain the effects of manuka essential oil on proliferation, normal human fibroblast cells (CUA-4) and fibrosarcoma cells (HT-1080) were plated into 24-well plates and then treated, while subconfluent, with increasing concentrations of manuka essential oil for 24 h. Cells were counted using the trypan blue dye exclusion technique and compared to untreated controls, as well as to vehicle controls (Figure 3). Manuka essential oil decreased cell proliferation in a dose-dependent manner in both cell lines. The vehicle control showed no significant decreases in the proliferation of the cells. The most significant decreases in proliferation were seen at a concentration of 500 µg/mL of manuka essential oil (a 94% decrease in CUA-4 fibroblast cells and a 55% decrease in HT-1080 fibrosarcoma cells). Similarly, a significant decrease (46%) was also seen in HeLa cervical adenocarcinoma cells [12].

**Figure 4:**
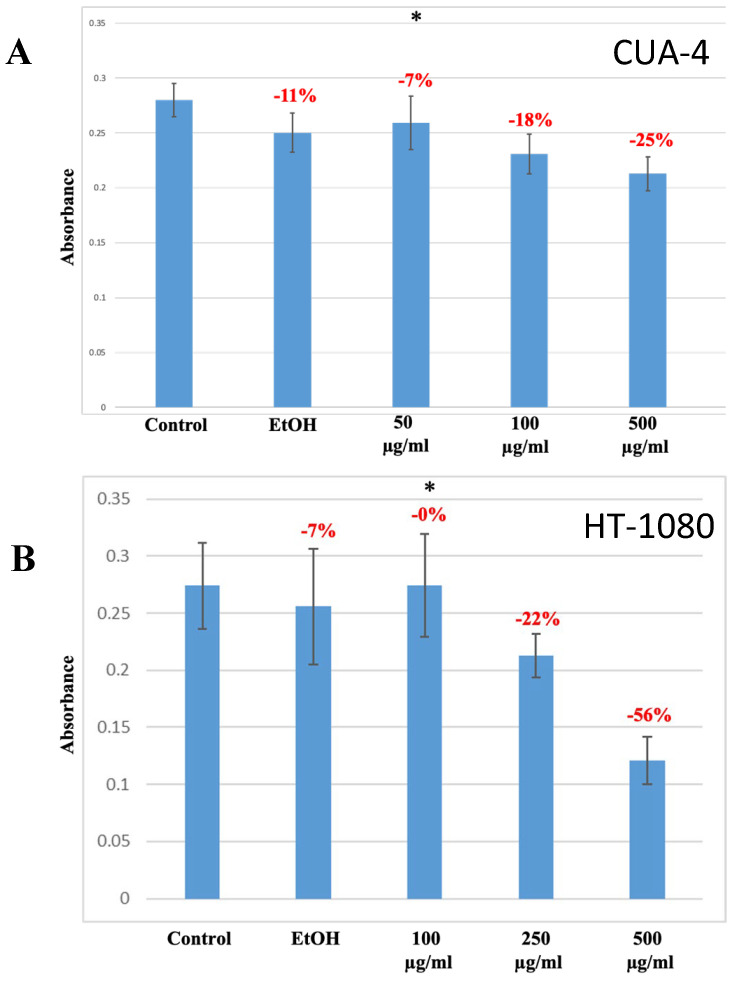
Manuka treatment decreased cell viability. The indicated cells were plated into 24-well plates, serum-starved overnight, and treated for 24 h with the indicated concentrations of manuka essential oil. An MTT assay was then performed to determine cell viability. Controls were left untreated (“control”) or were treated with ethanol (vehicle for oil dilution) (“EtOH”). (**A**) CUA-4 fibroblasts. * results were the average of 8 independent experiments and were found to be significant using ANOVA (α = 0.005). (**B**) HT-1080 fibrosarcoma cells. * results were the average of 10 independent experiments and were found to be significant using ANOVA (α = 0.05).

The decrease in the cell numbers seen upon treatment with manuka essential oil was reasoned to be due to cell-cycle arrest and a decrease in cell viability. To further evaluate viability, an MTT assay was employed. CUA-4 fibroblast cells and HT-1090 fibrosarcoma cells were plated into 24-well plates, serum-starved overnight, and treated for 24 h with increasing concentrations of manuka essential oil prior to being assayed with MTT (Figure 4). In comparison to the control samples, a significant decrease in cell viability was found at a concentration of 500 µg/mL of manuka essential oil (a 25% decrease in CUA-4 cells and a 56% decrease in HT-1080 cells). Interestingly, there was a greater decrease in cell viability in the HT-1080 fibrosarcoma cells compared to the CUA-4 fibroblast cells at the same concentration of manuka EO.

### 2.3. Manuka Essential Oil Stimulates Apoptosis

**Figure 5:**
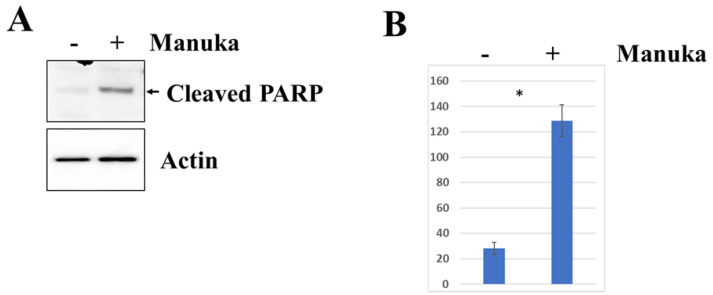
Manuka treatment causes the apoptosis of normal fibroblast cells. (**A**) CUA-4 normal fibroblast cells were plated into 100 mm plates, serum-starved overnight, and treated for 4 h with 1000 µg/mL of manuka essential oil. Controls were left untreated. Western blot analysis was performed using anti-PARP antibodies (Cell Signaling). Actin detection confirms equivalent loading. (**B**) The densitometry of the cleaved PARP bands. * results are the average of 11 independent experiments and were found to be significant using a *t* test (*p* < 0.10).

**Figure 6:**
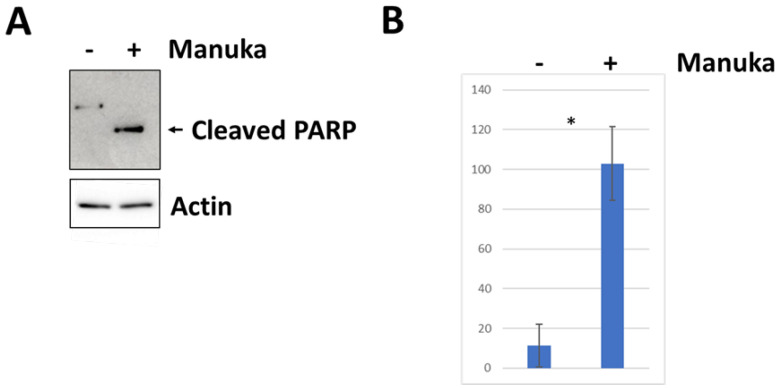
Manuka treatment causes the apoptosis of fibrosarcoma cells. (**A**) HT-1080 fibrosarcoma cells were plated into 100 mm plates, serum-starved overnight, and treated for 4 h with 1000 µg/mL of manuka essential oil. Controls were left untreated. Western blot analysis was performed using anti-PARP antibodies (Cell Signaling). The stained membrane and actin detection confirms equivalent loading. (**B**) The densitometry of the cleaved PARP bands. * results are the average of 10 independent experiments and were found to be significant using a *t* test (*p* < 0.05).

**Figure 7:**
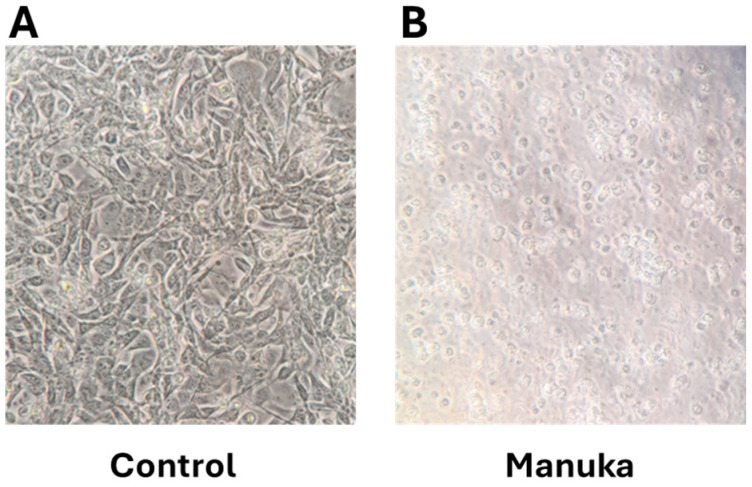
Manuka treatment causes morphology changes associated with late apoptosis in fibrosarcoma cells. HT-1080 fibrosarcoma cells were plated into 100 mm plates, serum-starved overnight, and treated for 24 h with 1000 µg/mL of manuka essential oil (**B**) or left untreated (control) (**A**). Manuka-treated cultures demonstrate smaller, rounded cells that demonstrate membrane blebbing.

Since proliferation and viability decreased in all cell lines due to the treatment of manuka essential oil, the ability of the oil to stimulate apoptosis was measured using the detection of the cleavage of poly(ADP-ribose) polymerase (PARP) [13]. Western blot analysis was used to detect cleaved PARP in cells treated for 4 h with 1000 µg/mL of manuka essential oil. Compared to untreated controls, CUA-4 fibroblast cells had a significant amount of cleaved PARP (Figure 5). Similarly, the cleaved PARP in HT-1080 fibrosarcoma cells significantly increased after treatment with manuka essential oil for 4 h (Figure 6). Actin detection was utilized to demonstrate equivalent loading. In addition to PARP cleavage serving as a marker for apoptosis, cell morphology changes occurred, with changes in cell size, shape, and membrane blebbing [14]. Furthermore, 24 h after manuka treatment of HT-1080 fibrosarcoma cells (Figure 7), these changes were also observed.

### 2.4. JNK Signaling Pathway Was Activated by Manuka Essential Oil

**Figure 8:**
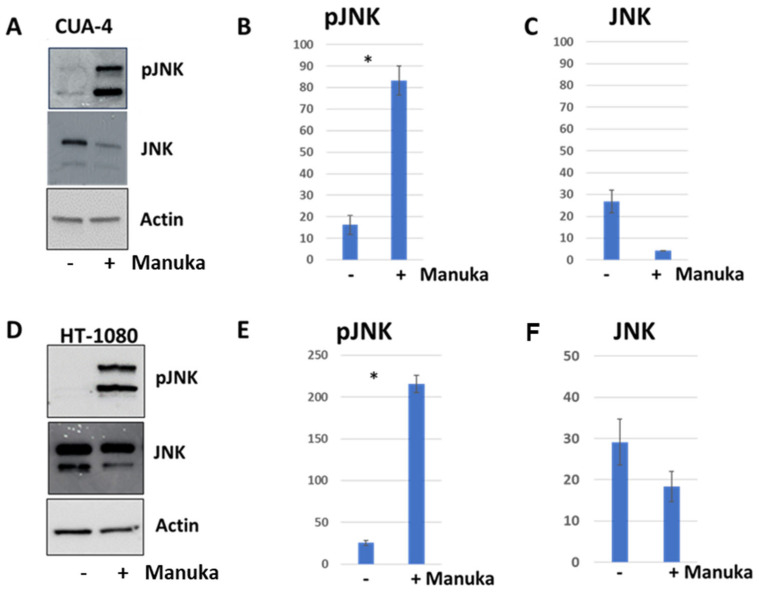
Manuka treatment increases the activity of JNK in normal human fibroblasts and fibrosarcoma cells. (**A**) CUA-4 normal fibroblast cells were plated into 100 mm dishes, serum-starved overnight, and treated for 4 h with the indicated concentrations of manuka essential oil. Western blot analysis was performed with the indicated antibodies to detect phosphorylated JNK, total JNK, and actin (loading control). Control cultures were left untreated (“control”). (**B**) The densitometry of phosphorylated JNK detection from CUA-4 cells. * results for pJNK detection are the average of 14 independent experiments and were found to be significant using a *t* test (*p* < 0.05). (**C**) The densitometry of JNK detection from CUA-4 cells. Results for JNK are representative of 6 independent experiments. (**D**) HT-1080 fibrosarcoma cells were plated into 100 mm dishes, serum-starved overnight, and treated for 4 h with the indicated concentrations of manuka essential oil. Western blot analysis was performed with the indicated antibodies to detect phosphorylated JNK, total JNK, and actin (loading control). Control cultures were left untreated (“control”). (**E**) The densitometry of phosphorylated JNK detection from HT-1080 cells. * results for pJNK detection are the average of 8 independent experiments and were found to be significant using a *t* test (*p* < 0.005). (**F**) The densitometry of JNK detection from HT-1080 cells. Results for JNK are representative of 14 independent experiments.

The mitogen-activated protein kinase c-Jun N-terminal kinase (JNK) can be activated by cellular stress. Therefore, the levels of active JNK were measured in both CUA-4 fibroblast cells and HT-1080 fibrosarcoma cells following a 4 h treatment with 1000 µg/mL manuka essential oil, while control cultures were left untreated. Western blot analysis was performed to detect phosphorylated JNK (pJNK) and total JNK. For both CUA-4 fibroblast cells and HT-1080 fibrosarcoma cells, the levels of pJNK were significantly increased in the cultures treated with manuka essential oil (Figure 8). There was no significant difference in the levels of total JNK present in both the treated and untreated cells. The blots were also re-probed with actin to demonstrate equivalent loading.

### 2.5. Manuka Essential Oil Differentially Regulates MAP Kinase Phosphatases

**Figure 9:**
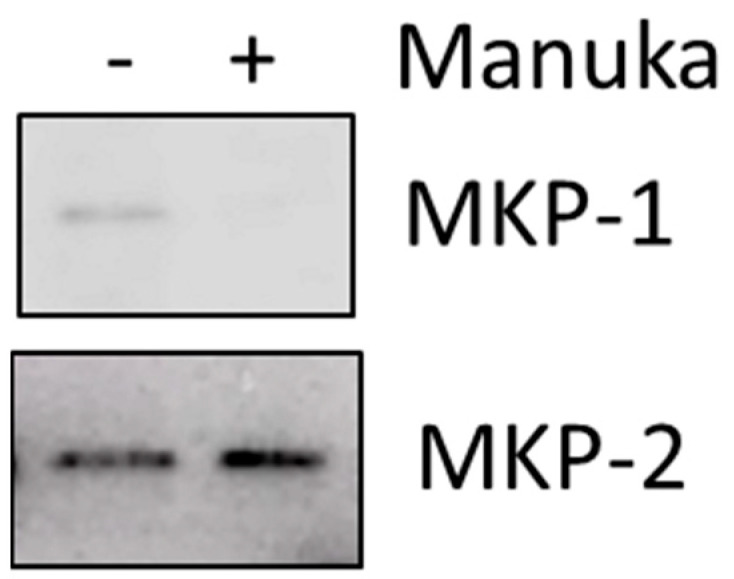
Differential expression of mitogen-activated protein kinase phosphatases (MKPs). HT-1080 fibrosarcoma cells were plated into 100 mm plates, serum-starved overnight, and treated for 4 h with 1000 μg/mL of manuka essential oil. Controls were left untreated. Western blot analysis was performed using anti-MKP1 (representative of two experiments) or anti-MKP2 antibodies (representative of three experiments) as indicated.

Dual-specificity mitogen-activated protein kinase phosphatases are a family of proteins which can negatively regulate the MAPK family through dephosphorylation. Since the level of activated JNK was altered by manuka essential oil, the expression of phosphatases that could regulate JNK was measured. HT-1080 fibrosarcoma cells were treated with manuka, and then Western blotting was used to detect both MKP-1 and MKP-2. MKP-1 expression was low in untreated cultures, but further decreased upon treatment with manuka essential oil. Conversely, MKP-2 expression increased in fibrosarcoma cells treated with manuka essential oil (Figure 9).

## 3. Discussion

There has always been an interest in the use of natural products for health and wellness. The movement for “green chemistry” and the increased need for new cancer treatments has renewed the search for alternative natural remedies for illness. The properties of manuka essential oil have led to its use in a variety of applications, including antibiotic, anti-viral, and anti-inflammation applications [4]. Many studies related to manuka, however, are focused upon manuka honey, which is produced by the bees that pollinate manuka trees; less is known regarding the uses of the essential oils from this tree [4]. Some research has been performed on the antimicrobial effects of manuka essential oil, though studies of the effects of manuka essential oil on eukaryotic cells, and on cancerous cell lines in particular, are limited. The need for additional anti-cancer agents is evident and, with their historical use in traditional medicine, essential oils may be a likely source. It is estimated that approximately half of the conventional chemotherapy agents are derived from plants or chemically modified plant products [15,16]. Some essential oils have been shown to have anti-tumor activities. For example, camphene, isolated from the essential oil of *Piper cernuum*, caused the apoptosis of melanoma cells through increasing endoplasmic reticulum (ER) stress [17]. *Melissa officinalis* EO was found to induce the apoptosis of glioblastoma cells through a reactive oxygen species (ROS)-dependent mechanism [18]. The pathways for the induction of apoptosis may be unique for each EO and cell type.

As an essential oil, manuka oil is composed of multiple constituents, which may all act synergistically to produce the biological effects. Likewise, the combination of this particular oil with other natural products may result in collaborative effects. The understanding of the network pharmacology influenced by this treatment would be a beneficial goal for future work [19]. Manuka oil contains the same constituents as most essential oils, including monoterpenes, sesquiterpenes, and eudesmols. The triketone complex, containing isoleptospermone, leptospermone, and flavesone, is most desirable as leptospermone has been found to have many medicinal properties [20]. As reviewed by Sharma et al. [21], EOs have antioxidant, antiproliferative, and anti-mutagenic characteristics, which make them ideal potential sources for cancer treatments. Leptospermone is a *p*-hydroxyphenylpyruvate dioxygenase (HPPD) inhibitor. HPPD breaks down excess tyrosine that builds up in plant and animal cells and, if there is such a buildup of tyrosine, the cells will undergo death from oxidative stress [22]. A chloroform extract of *L. scoparium*, containing the major constituents cis-calamenene, beta-eudesmol, cyclododecane, and alpha-muurolene, was recently found to have cytotoxic effects on breast and liver cancer cells [23].

The HT-1080 cell line used in this study is a fibrosarcoma cell line, bearing an NRAS mutation, and also expressing high levels of MMP-9, which contributes to its invasiveness [24]. This cell line has been used to study the effects of natural products on proliferation and survival. Furanodiene, an essential oil component extracted from *Curcuma wenyujin*, was shown to inhibit the proliferation of HT-1080 fibrosarcoma cells [25]. Likewise, decreased proliferation and apoptosis occurred upon the treatment of HT-1080 cells and normal human fibroblasts (CUA-4) with kumquat essential oil [26]. The results of all cell lines tested were similar—manuka EO decreased proliferation in both cell lines as measured through trypan blue dye exclusion (Figure 3). This decrease was dose-dependent and was not caused by the ethanol used as the vehicle for treatment. Comparably, the viability of fibrosarcoma cells and normal human fibroblasts was also tested using an MTT assay. Though some limitations have been noted for the use of the MTT assay to measure viability [27], the assay does measure metabolic activity consistent with cell survival; cell density and treatment duration were kept constant. The results of the MTT assay of manuka-treated cells mirrored the results seen from counting cells with trypan blue dye exclusion, as there was a dose-dependent decrease in viability (Figure 4). For the normal human fibroblasts, the decrease in viability detected with MTT at the highest concentration of manuka EO used (500 µg/mL) was less of a decrease from the control (−25%) than what was detected from cell counting with trypan blue dye exclusion (−94%). Confluent fibroblasts have been shown to be more resistant to apoptosis [28]), so this could be a reflection of confluency slowing the metabolic response to the manuka EO. Additionally, trypan blue dye exclusion, like the MTT assay, also has some limitations, and the results are assessed subjectively [29]. This technique is measuring membrane integrity, which may be compromised more quickly than the cellular metabolism due to the essential oil treatment. It is also possible for cell injury to occur during the assay, which may lead to more dye uptake.

The decreased viability in cultures treated with manuka EO, and their altered morphology, suggested that the treated cells were experiencing apoptosis. The levels of cleaved PARP were measured in order to determine if apoptosis was happening as the result of this treatment. In both fibrosarcoma cells and normal fibroblasts, treatment with manuka EO led to increased PARP cleavage (Figure 5 and Figure 6), confirming apoptosis in both cell lines. In addition to PARP cleavage, changes in cell morphology were also observed (Figure 7). This study is limited to these late-stage methods of apoptosis detection. The JNK mitogen-activated protein kinase (MAPK) is a stress-regulated protein, which may have a role in activating pro-apoptotic genes [30]. In both normal fibroblasts and fibrosarcoma cells, manuka EO caused a significant increase in the phosphorylation of JNK (Figure 6). In correlation, the activation of JNK has been implicated in apoptosis of fibroblasts and fibrosarcoma cells following treatment with kumquat EO [26], and phosphorylation of both p38 and JNK MAPKs occurred following the treatment of MCF-7 cells with the natural plant product bornyl caffeate, leading to apoptosis [31]. The JNK pathway can be negatively regulated by the phosphatases MKP-1 and MKP-2, so the levels of these two proteins were detected in fibrosarcoma cells treated with manuka EO (Figure 9), and these proteins appear to be differentially regulated by manuka treatment. MKP-1 expression, though detected in low amounts in untreated cells, further decreased upon manuka EO treatment. The decrease in MKP-1 correlated with an increase in the phosphorylation of JNK. However, MKP-2 expression was slightly increased by manuka EO treatment. MKPs are important regulators of the JNK pathway and can be activated by JNK signaling as well [32], resulting in a feedback loop. It is possible that the increased activation of JNK can also cause a transient increase in MKP-2. Taken together, these results suggest that manuka EO treatment causes apoptosis through a JNK-regulated pathway.

## 4. Materials and Methods

### 4.1. Cell Lines and Culture Conditions

HT-1080 fibrosarcoma cells and HeLa cells were purchased from ATCC (Manassas, VA, USA). CUA-4 fibroblast cells were a gift from Dr. James Greene (The Catholic University of America, Washington, DC, USA). All cell lines were maintained in Dulbecco’s Modified Eagle’s Medium (DMEM) (Life Technologies, Carlsbad, CA, USA), supplemented with 10% fetal bovine serum (R&D Systems, Minneapolis, MN, USA) at 37 °C in a humidified atmosphere containing 5% CO_2_. Manuka essential oil was purchased from dōTERRA (Pleasant Grove, UT, USA). The essential oil was extracted from *Leptospermum scoparium* shrubs in New Zealand. The manuka EO was first diluted in 95% ethanol to make a concentrated stock solution (100,000 μg/mL), and then further diluted in PBS to create a 10,000 μg/mL stock solution prior to being added to a culture medium at the indicated concentrations. A single lot of manuka EO was utilized for all experiments. The photography of cells was accomplished using a Nikon Eclipse inverted microscope (200×).

### 4.2. Gas Chromatography–Mass Spectrometry (GC-MS)

Manuka essential oil was analyzed by GC-MS using a Shimadzu GCMS-QP2010S (Shimadzu Scientific Instruments, Columbia, MD, USA) operated in the electron impact (EI) mode (electron energy = 70 eV), scan range = 40–400 atomic mass units, scan rate = 3.0 scans/s, and GC-MS solution software (ver 4.45). The GC column was a ZB-5 fused silica capillary column with a (5% phenyl)-polymethylsiloxane stationary phase and a film thickness of 0.25 μm. The carrier gas was helium with a column head pressure of 552 kPa and flow rate of 1.37 mL/min. The injector temperature was 250 °C and the ion source temperature was 200 °C. The GC oven temperature program was programmed for 50 °C initial temperature; temperature increased at a rate of 2 °C/min to 260 °C. A 5% w/v solution of the sample in CH_2_Cl_2_ was prepared and 0.1 μL was injected with a splitting mode (30:1). The identification of the oil components was based on their retention indices determined by reference to a homologous series of n-alkanes, and by comparison of their mass spectral fragmentation patterns with those reported in the literature [33];they were stored in our in-house MS library.

### 4.3. Proliferation Assays

We plated 6.0 × 10^7^ cells per well of 24-well plates. Some 24 h after plating, cells were treated with manuka essential oil, with ethanol (control), or were left untreated for 24 h. Direct counting with a hemocytometer and trypan blue dye exclusion was used to determine proliferative capacity. Viability was assessed through plating the cells in 24-well plates, serum-starving overnight, then treating the cells with the indicated concentrations of manuka EO or ethanol (vehicle control) for 24 h in phenol red-free culture medium, followed by treatment with MTT (3-(4,5-dimethylthiazol-2-yl)-2,5-diphenyltetrazolium bromide). The MTT assay was performed as per the protocol of the kit manufacturer (Cell Growth Determination Kit, Sigma, St. Louis, MO, USA).

### 4.4. Western Blot Analysis

The indicated cell lines were plated into 100 mm plates, serum-starved overnight, and treated for 4 h with 1000 μg/mL of manuka EO. Controls were left untreated. Western blot analysis was performed as previously described [34]. Briefly, cell cultures were washed twice with ice-cold phosphate-buffered saline (PBS) and then lysed in 1000 µL of lysis buffer (20 mM N-2-hydroxyethylpiperazine-N^1^-2-ethane sulfonic acid (HEPES), pH 7.4, 50 mM β-glycerophosphate, 1% Triton X-100, 10% glycerol, 2 mM ethylene bis (oxyethylenenitrilo) tetraacetic acid (EGTA), 1 mM DTT, 10 mM sodium fluoride, 1 mM sodium orthovanadate, 2 μM leupeptin, 2 μM aprotinin, and 1 mM phenylmethylsulfonyl fluoride (PMSF). The lysates were clarified by centrifugation at 14,000 rpm for 10 min. Then, 30 μg of each protein sample was resolved by electrophoresis through 10% Tris-Glycine gels (NuSep, Germantown, MD, USA). Anti-PARP (9532), anti-p-SAPK/JNK (T183/Y185) (9255), and anti-SAPK/JNK (9252) antibodies were purchased from Cell Signaling Technologies (Danvers, MA, USA). Anti-actin (sc-517582) antibodies were purchased from Santa Cruz Biotechnology, Inc. (Santa Cruz, CA, USA), and anti-MKP-2 antibodies were purchased from BD Transduction Laboratories (San Jose, CA, USA). The Clarity Western ECL substrate chemiluminescence reagent (BioRad, Hercules, CA, USA) was used for the detection of the immunoreactive bands. Densitometry was performed on reactive bands utilizing the public domain NIH Image program (developed at the U.S. National Institutes of Health and available on the Internet at ImageJ (URL imagej.net/nih-image (accessed on 4 April 2024)).

## Figures and Tables

**Table 1:** Manuka essential oil constituents. Typical R time and area% for each component are indicated.

R.Time.	Name	Area %
12.746	Pinene <alpha>	0.74
15.263	Pinene <beta>	0.09
15.898	Myrcene	0.22
18.141	Cymene <para>	0.07
18.440	Limonene	0.05
18.619	1,8-cineole	0.10
20.329	Terpinene <gamma>	0.09
23.529	Isopentylisovalerate	0.08
24.187	Methyl butanoate 3-methyt-3-buteryl <3-iso>	0.12
39.935	Cubebene <alpha>	3.51
41.385	Ylangene <alpha>	0.12
41.818	Copaene <alpha>	4.00
42.585	Cubebene <beta>	0.09
42.696	Elemene <beta>	1.05
43.021	Isoovalerate <benzyl>	0.14
43.827	Gujunene <alpha>	0.76
44.621	Caryophylene <trans>	2.26
45.274	Copaene <beta>	0.20
45.806	Aromadendrene	1.28
45.979	Guaiadiene <6,9->	0.12
46.254	Manuka	0.17
46.489	Murrola-3,5-diene <trans>	6.95
46.868	Humulene <alpha>	0.28
47.146	Alloaromadendrene	0.52
47.901	Cadina-1(6).4-diene <cis>	3.45
48.076	Cadilna-1(6).4-diene <trans>	0.78
48.198	Gurjunene <gamma>	0.10
48.337	Amorphene <alpha>	0.17
48.458	Germacrene D	0.18
48.970	Selinene <beta>	4.03
49.129	Muurda-4(14),5-diene <trans>	1.94
49.401	Selinene <alpha>	3.94
49.526	Muurdene <alpha>	0.71
49.885	Farnesene <(E.E)alpha>	1.27
50.419	Amorphene <delta>	0.58
50.754	Cadinene <delta>	4.28
50.976	Calamenene <trans.>	11.56
51.602	Cadine-1.4-diene <trans->	5.23
51.820	Flavesone	6.46
52.092	Calacorene <alpha>	0.47
53.335	Calcorene <beta>	0.11
53.540	Manuka	0.09
54.193	Spathulenol	0.48
54.509	Caryophylene axide	0.38
54.738	Globulol	0.41
55.828	Lidol	0.16
56.199	Leptospermone <iso>	6.15
56.572	Intermedeolisomer	0.10
56.828	Leptospermone	19.52
56.955	Eudesmol <isomer>	0.11
57.168	Cubenol- <1-epi>	0.94
57.450	Manuka	0.09
58.015	Cubanol	1.53
58.207	Cadinol <delta>	0.11
58.709	Eudesmol <alpha>	0.40
58.874	Selin-11-en-4-alpha-ol	0.30
59.678	Cadalene	0.13
60.003	Copaene-4beta-ol	0.07
62.619	Muurol-5en-4one Isomer	0.10
84.005	Grandiflorone	0.68
		100.00

## Data Availability

Data are available upon request from the corresponding author.
